# Acute hydrothorax complicating peritoneal dialysis: a case report

**DOI:** 10.1186/1752-1947-4-355

**Published:** 2010-11-08

**Authors:** Yeoungjee Cho, Vincent D'Intini, Dwarkanathan Ranganathan

**Affiliations:** 1 Renal Unit, Royal Brisbane & Women's Hospital, Level 9 Ned Hanlon Building, Bowen Bridge Road, Herston, Brisbane, Queensland, 4029, Australia; 2 School of Medicine, The University of Queensland, Queensland, 4072, Australia

## Abstract

**Introduction:**

Acute hydrothorax is an uncommon but a well-recognized complication of peritoneal dialysis. No single test is definitive for diagnosis. Although it is not a life-threatening condition, hydrothorax often requires abandonment of peritoneal dialysis. Delay in diagnosis can lead to worsening of the clinical status.

**Case Presentation:**

A 33-year-old Caucasian woman with lupus, who was successfully treated with temporary peritoneal dialysis 17 years previously, presented with acute dyspnea and a right pleural effusion after recommencing peritoneal dialysis. Investigations eliminated infective, cardiac, and primary respiratory causes. Peritoneal dialysis-related hydrothorax was suggested by biochemistry, and a pleuroperitoneal leak was definitively confirmed by using a Tc-99 m DTPA (diethylene triamine penta-acetic acid) scintigraphy scan. Subsequently, she underwent video-assisted thoracoscopy-guided talc pleurodesis and was able to return successfully to peritoneal dialysis.

**Conclusion:**

Although our case is not the first report that describes the occurrence of acute hydrothorax in peritoneal dialysis, it is an important condition to recognize for the wider general medical community. Furthermore, this case demonstrates that peritoneal dialysis can be continued with a hydrothorax, provided the underlying cause can be corrected. We review the literature pertaining to the utility and reliability of different diagnostic approaches to hydrothorax.

## Introduction

Peritoneal dialysis-(PD) related hydrothorax was first reported in 1967 by Edward and Unger [1]. The prevalence of hydrothorax varies, ranging from 1.6% [2] to 6% of adult PD patients [3]. Transudative pleural effusion develops, more commonly involving the right side, and usually occurs immediately after starting PD or a few days later [4]. The patients may remain asymptomatic or have sudden dyspnea, decrease in ultrafiltration, or pleuritic chest pain.

Possible pathogenetic mechanisms include congenital diaphragmatic defects, a disorder of lymphatic drainage, and pleuroperitoneal pressure gradient [4-6]. The theory of congenital diaphragmatic defects explains the preponderance of right-sided hydrothorax because left-sided defects, as such, are covered by the heart and pericardium, thereby protecting against the leak. Reported risk factors for developing hydrothorax include PD peritonitis; it may exacerbate the continuity defects in the pleuroperitoneal structure [7]. However, transudative pleural effusion can be due to multiple causes other than PD, such as congestive heart failure, hypoalbuminemia, or fluid overload for any reason.

An accurate method of diagnosing pleuroperitoneal communication is important, as treatment efforts using hypertonic peritoneal exchanges to improve fluid status may worsen the hydrothorax [8]. Unfortunately, no single definitive test exists for the diagnosis and localization of the defect for potential intervention.

## Case presentation

A 33-year-old Caucasian woman with end-stage renal failure secondary to systemic lupus erythematosus started PD in December 2008. She had previously been treated with PD without complication 17 years before, in the context of acute kidney injury, from which she recovered. Exact details of PD were unavailable, but it was recalled to be of short duration and probably of small fill volume. The patient denied any history of traumatic injury to the chest or diaphragmatic surgery.

A Tenckhoff catheter was inserted 28 days before commencement of PD without complication. Her first exchange revealed asymptomatic slightly cloudy dialysate with 2^10 ^white cells [82% polymorphonuclear cells] and negative growth and was treated as culture-negative PD peritonitis.

**Figure 1:**
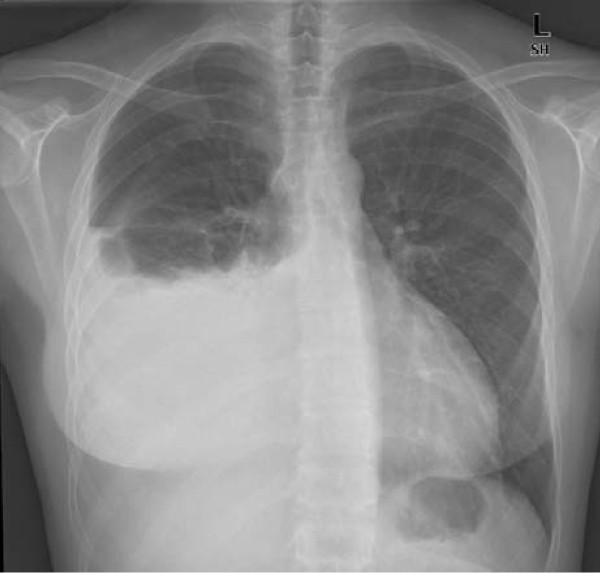
**Chest X-ray showing right pleural effusion**.

One week later, she presented with sudden onset of dyspnea associated with right pleuritic chest pain. Chest X-ray (Figure 1) showed right pleural effusion.

Thoracentesis was performed for symptomatic relief. Pleural fluid biochemistry results were consistent with transudate, according to Light's criteria (Table 1). Culture of pleural aspirate showed no evidence of infection. Clinically, no evidence of lupus flare or nephrotic syndrome was noted.

**Table 1:** Serum, peritoneal, and pleural fluid biochemistry

Fluid type	Glucose [mmol/L]	Protein [g/L]	Lactate dehydrogenase
Serum	5.1	71	282
Peritoneal	31.1	< 5	8
Pleural	12.5	< 5	28

**Figure 2:**
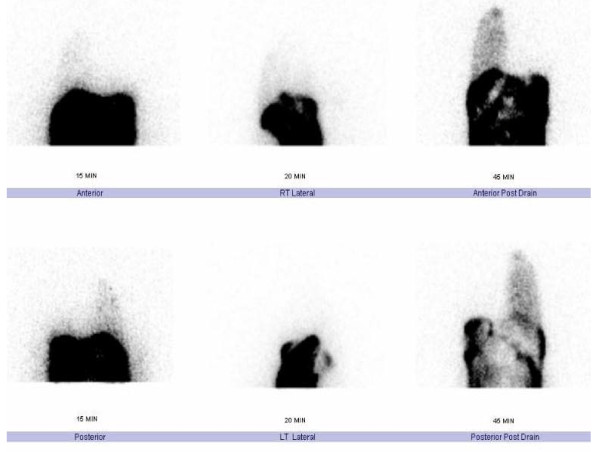
**Tc-99 m DTPA scintigraphy showing pleuroperitoneal leakage**.

Pleural fluid biochemistry results were consistent with PD-related hydrothorax. An effusion reaccumulated with further PD treatment. It was unclear whether poor dialysis (uremia) or ultrafiltration was contributing to the effusion. Tc-99 m DTPA scintigraphy was performed to confirm pleuroperitoneal leakage, showing prompt (within 15 minutes) appearance of tracer in the pleural space on the right side with no evidence of tracer entering the left hemithorax (Figure 2). Thus, the etiology of hydrothorax likely was due to congenital diaphragmatic defects, exacerbated by persistently elevated intraabdominal pressure from PD.

The patient was temporarily transferred to hemodialysis via a tunneled line and underwent right video-assisted thoracoscopic surgery and talc pleurodesis. An intraoperative methylene blue test failed to identify the pleuroperitoneal defect. Two months after the procedure, she successfully recommenced PD without reoccurrence of hydrothorax.

## Discussion

Diagnostic approaches to PD-related hydrothorax can be broadly divided into biochemical and imaging approaches.

The basis of the biochemical approach is that PD-related hydrothorax is believed to have a high pleural-fluid glucose concentration. However no validated reference range exists to aid diagnosis. Some investigators believe that only a pleural fluid glucose concentration greater than 16.5 mmol/L (300 mg/dl) would be diagnostic [9].

Chow and others [10] have hypothesized that, given dynamic movement of dialysate, an absolute glucose-concentration level cannot be used to diagnose PD-related hydrothorax. The pleural fluid-to-serum glucose concentration gradient of greater than 2.77 mmol/L (50 mg/dl) was proposed as the cut-off to diagnose the condition [10]. Subsequent studies, however, found this to be unreliable [11], and generally any pleural-fluid glucose concentration greater than serum is considered to be highly supportive of PD-related hydrothorax.

All studies assessing imaging techniques have very small subject numbers, and none of the test methods is very sensitive. Radionuclide scan (for example, Tc-99 m DTPA) is associated with sensitivity of 40% to 50% [11,12]. A case report by Ortiz and others [13] showed that the yield of scintigraphy in a child with hydrothorax could be improved if the scanning were performed immediately after complete drainage of the hydrothorax, possibly because of relief of this pressure.

The methylene blue test in one study showed no sensitivity and is associated with a risk of chemical peritonitis [11]. Contrast computed tomography peritoneography was associated with 33% sensitivity in one study, with added risk of nephrotoxicity in those with residual renal function [11,12].

## Conclusion

PD-related hydrothorax is an important complication of PD to be recognized and treated in a timely fashion. Current diagnostic strategies to confirm PD-related hydrothorax are unsatisfactory. Pleural-fluid glucose concentration that is greater than that of serum is most probably due to pleuroperitoneal leak. Methods to improve the sensitivity of a radionuclide scan should be studied, such as thoracentesis to decrease intrathoracic pressure. More important, it is imperative to recognize that PD hydrothorax does not have to lead to abandonment of PD as long as the etiology can be corrected.

## Competing interests

The authors declare that they have no competing interests.

## Authors' contributions

All authors contributed to writing and reviewing of the manuscript.

## Consent

Written informed consent was obtained from the patient for publication of the case report and accompanying images. A copy of the written consent is available for review by the Editor-in-Chief of this journal.
